# Predictors Associated with Outcomes of Epidural Blood Patch in Patients with Spontaneous Intracranial Hypotension

**DOI:** 10.3390/jcm10050922

**Published:** 2021-02-28

**Authors:** Jun-Young Park, Young-Jin Ro, Jeong-Gil Leem, Jin-Woo Shin, Yul Oh, Seong-Soo Choi

**Affiliations:** 1 Department of Anesthesiology and Pain Medicine, Asan Medical Center, University of Ulsan College of Medicine, Seoul 05505, Korea; parkjy@amc.seoul.kr (J.-Y.P.); thisisyjro@naver.com (Y.-J.R.); jgleem@amc.seoul.kr (J.-G.L.); sjinwoo@hotmail.com (J.-W.S.); 2 Spine Center, Namyangju Baek Hospital, Namyangju-si 12066, Korea

**Keywords:** spontaneous intracranial hypotension, epidural blood patch, neutrophil-to-lymphocyte ratio

## Abstract

An autologous epidural blood patch (EBP) is a mainstay of treatment in patients with spontaneous intracranial hypotension (SIH). EBP, however, is less effective for SIH than post-dural puncture headaches. Therefore, patients with SIH frequently require an additional EBP. The aim of this study was to identify factors associated with poor response to EBP. This single-center retrospective observational study used the institutional registry records of 321 patients who underwent EBP between September 2001 and March 2016. Patients were divided into two groups, a poor responder group, consisting of patients who underwent EBP at least three times or more, and a good responder group of patients who experienced sufficient symptom relief after two or fewer EBP. The demographic characteristics, clinical features, radiologic findings, procedural data, and laboratory data were analyzed. Univariate analysis showed that the neutrophil-to-lymphocyte ratio (NLR; *p* = 0.004) and platelet-to-lymphocyte ratio (*p* = 0.015) were significantly lower in poor than in good responders. Multivariate analysis found that NLR was the only independent factor associated with a poor response (odds ratio = 0.720; *p* = 0.008). These findings indicate that a low NLR was associated with three or more EBP administrations for the sufficient improvement of symptoms in patients with SIH.

## 1. Introduction

Spontaneous intracranial hypotension (SIH) manifests as various symptoms due to low cerebrospinal fluid (CSF) pressure [1,2]. The most common symptom is an orthostatic headache, which may be accompanied by other symptoms, such as nausea, vomiting, auditory problems, facial numbness, and visual problems, with or without neurologic deficits [3,4]. Several studies have attempted to determine the etiologies and treatments for spontaneous spinal fluid leakage [5]. According to the results of these studies, a non-surgical treatment including epidural blood patch (EBP) showed acceptable outcomes [6,7]. EBP is a widely performed method of interventional management to the level of the leakage site or, when leakage level is not determined, to the level of the provisional leakage site.

Previous clinical studies have shown that EBP is an acceptable SIH treatment despite the variability in its success rates [8]. Patients who do not respond adequately to the first EBP should undergo repeated EBP. At least one-fourth of patients with SIH are not cured by a single EBP, and about 50% require more than one EBP [8,9]. In addition, EBP is less effective in treating SIH than post-dural puncture headaches (PDPHs) [10,11,12,13]. To date, guidelines regarding when and how to attempt additional EBP have not been determined. Therefore, identifying the factors associated with the response to EBP is important for the evaluation and prediction of prognosis in patients with SIH.

Several factors are thought to predict a poor response to EBP in patients with SIH [11,14], including early bladder activity on radioisotope cisternography, severe spinal CSF leakage, a diencephalic–mesencephalic deformity on magnetic resonance imaging (MRI), and an injected blood volume less than 22.5 mL. To date, however, factors associated with the treatment outcomes of EBP for SIH have not been fully evaluated. Furthermore, previous studies focused primarily on radiologic findings and interventional characteristics, not on biological or biochemical markers. The present study therefore sought to identify the factors associated with a poor response to EBP, including clinical features, radiologic findings, procedural characteristics of EBP, and laboratory findings, in patients with SIH. This evaluation may help to estimate the responses to EBP and determine guidelines for EBP use in the treatment of SIH.

## 2. Materials and Methods

This single-center retrospective observational study evaluated the institutional registry records of 321 patients who underwent EBP for SIH between September 2001 and March 2016. The study protocol was approved by the ethics board of our institution (approval number, 2019–0832), which waived the requirement for obtaining informed consent due to the retrospective nature of this study.

### 2.1. Patients

Patients were included if (1) they were diagnosed with SIH by the neurology department, (2) they underwent autologous EBP, (3) both the radioisotope cisternography and brain MRI results were available, and (4) they were discharged with significant symptom improvement. Patients were excluded if (1) their medical records were incomplete, or (2) there were no records of a radioisotope cisternography or brain MRI. Each patient was initially managed with supportive treatment by a neurologist. The diagnosis of SIH was established by a neurologist according to the Headache Classification Committee of the International Headache criteria for headache attributed to SIH (7.2.3) [15,16]. If these initial supportive treatments failed within 1 week, patients were referred to the pain clinic, and an autologous EBP was performed by an experienced pain clinician.

### 2.2. Demographic, Clinical Characteristics, and Neuroimaging

Patients’ electronic medical records were reviewed, and factors such as age, sex, height, weight, body mass index (BMI), and underlying diseases, such as diabetes mellitus, hypertension, coronary arterial disease, cerebrovascular disease, and herniated intervertebral disc, were analyzed.

A history of headaches such as migraines, tension headaches, and cluster headaches, and associated symptoms such as nausea, vomiting, photophobia, hearing impairment, tinnitus, vertigo, or diplopia, duration of headache, and headache intensity using an 11 point (0–10) numeric rating scale were collected and analyzed from the medical records.

A brain MRI, radioisotope cisternography, and the measurement of the CSF opening pressure were performed prior to EBP. The brain MRI findings were reviewed by the radiologists. The flowing signs included pachymeningeal enhancement [17], engorgement of venous structures [18], brain sagging [19], pituitary hyperemia [18], midline shift [3], midbrain-pons angle [20], and the vein of the Galen-Straight sinus angle [21]. The midbrain–pons angle was defined as the angle between the line tangential to the anterior margin of the midbrain and the line tangential to the superior margin of the pons on the sagittal midline of the brain MRI. The angle between the vein of Galen and the straight sinus was regarded as an index of downward stretching.

The results of the radioisotope cisternography were reviewed to determine the location of the leakage and the number of leakage levels. The leakage levels were classified according to the spine anatomy and sorted into three types: cervical, thoracic, and lumbar [22]. The cerebrospinal opening pressure and early bladder activity were also reviewed before EBP. Early bladder activity was indicated by the presence of radioactivity in the urinary bladder 1 to 3 h after a lumbar intrathecal injection of a radioactive tracer [14].

### 2.3. Epidural Blood Patch

A targeted autologous EBP was performed using a 21-gauge Tuohy needle via a midline or paramedian approach under fluoroscopic guidance with a C-arm system (OEC 9800, General Electric Healthcare, Little Chalfont, United Kingdom) with the patient in the prone position. The epidural space was identified using the loss of resistance technique, and accurate localization was confirmed by ensuring the spread of the injected contrast medium over the targeted epidural space. Thereafter, autologous blood was slowly injected until the patient began to appeal any back or radicular pain. The targeted volume of venous blood injection was 20 mL; however, the epidural administration was stopped when patients started to feel any discomfort during the procedure even if the target volume was not reached.

The target level of autologous EBP and the level of most increased paraspinal activity were determined by radioisotope MRI cisternography. In case of an undetermined site of leakage, EBP at the lumbar level was performed. If complete remission did not occur within 72 h, an additional autologous EBP was delivered. In patients with multiple leakage sites, the second EBP was performed to a level different to the first, whereas in patients with single leakage sites, the second EBP was delivered to the same target level. EBP was repeated until full resolution of symptoms. The number of total EBPs, the volume of the first EBP, and site of first EBP were analyzed.

### 2.4. Laboratory Data

Laboratory tests were conducted one day before EBP. Laboratory tests included hemoglobin, hematocrit, platelet, prothrombin time, platelet distribution width, activated partial thromboplastin time, white blood cell count, absolute neutrophil count, and C-reactive protein. The percentage of neutrophil, lymphocyte, monocyte, eosinophil, and basophil were also performed. The neutrophil-to-lymphocyte ratio was calculated by the ratio of the absolute neutrophil counts and the absolute lymphocyte counts. The platelet-to-lymphocyte ratio was calculated by the ratio of the absolute platelet counts and absolute lymphocyte counts; the platelet-to-neutrophil ratio was calculated by the ratio of the absolute platelet counts and absolute neutrophil counts.

### 2.5. Hospitalization Period

Patients were hospitalized until all their symptoms were fully resolved. The hospitalization period was defined as the period from the day of admission for SIH to the day of discharge without other complications.

### 2.6. Treatment Response

Complete remission was defined as a resolution of all symptoms within 72 h after EBP, persisting for at least 3 months [11,23]. Patients who continued to experience symptoms underwent repeated EBP just after their response to the preceding EBP was assessed, with a successful response to repeated EBP assessed as a resolution of symptoms within 72 h.

Various studies have reported that patients with SIH may have to receive EBP twice or more times for the sufficient improvement of symptoms [9,13,24,25]. In this study, therefore, patients who required three or more EBPs for the sufficient improvement of symptoms were categorized as poor responders, whereas those who had sufficiently improved their symptoms with one or two EBPs were categorized as good responders.

### 2.7. Statistical Analysis

The continuous variables are presented as means and standard deviations or, if skewed, as medians and interquartile ranges. The categorical variables are presented as absolute numbers and percentages. The continuous variables were compared using the Student’s t-test or the Mann-Whitney U test, as appropriate. Categorical data were compared using the chi-square test. The most relevant factors associated with a response to EBP were included in a univariate logistic regression analysis. The inclusion of variables in the final multivariate logistic regression analysis evaluating factors independently associated with a poor response was based on biological plausibility, clinical importance and statistical considerations (*p* < 0.10). All statistical analyses were performed using SPSS 21.0.0 for Windows (SPSS Inc., Chicago, IL, USA), with two-tailed *P* values <0.05 considered statistically significant.

## 3. Results

**Figure 1:**
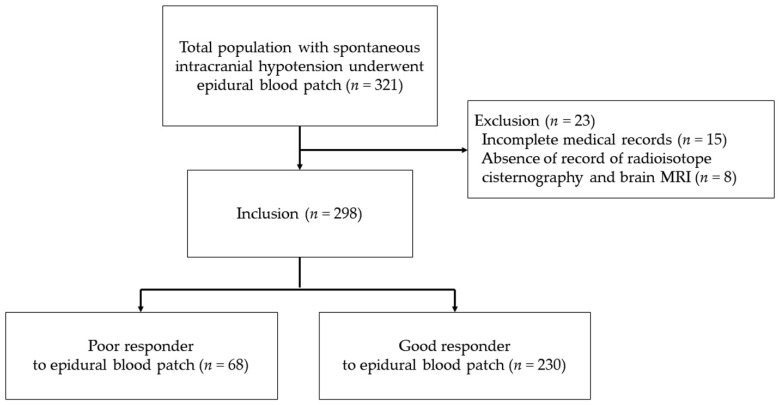
Flow diagram of the study. Poor responders were defined as patients who underwent epidural blood patch at least three times. Good responders were defined as patients who underwent epidural blood patch one or two times.

From September 2001 to March 2016, 321 inpatients at our institution were diagnosed with SIH and underwent EBP. Of these, 23 patients were excluded from analysis, 15 patients were excluded due to incomplete medical records and eight patients were excluded because of the absence of records of a radioisotope cisternography and brain MRI. In total, 298 patients fulfilled both the inclusion and exclusion criteria and were included in this analysis (Figure 1).

### 3.1. Demographic and Clinical Characterstics

The baseline patient demographic characteristics are shown in Table 1. Of the 298 patients, 68 (22.8%) were categorized as poor responders, requiring three or more EBPs, and 230 (77.2%) as good responders, requiring one or two EBPs. The baseline characteristics of the two groups were similar except for hypertension (Table 1), which was fewer in poor than in good responders (0 (0.0%) vs. 15 (6.5%), *p* = 0.027). Age, sex, height, weight, BMI, diabetes mellitus, coronary arterial disease, cerebrovascular accident, herniated intervertebral disc, migraines, tension headaches, cluster headaches, and associated symptoms such as nausea, vomiting, photophobia, hearing impairment, tinnitus, vertigo, and diplopia, duration of headache, and severity of headache did not differ significantly in the two groups.

### 3.2. Neuroimaging

The most common abnormality on the brain MRI was pachymeningeal enhancement (*n* = 161 (54.0%)) (Table 2). None of the brain MRI signs differed between the groups of poor and good responders. The rate of engorgement of venous structures was slightly higher in poor than in good responders, but the difference was not statistically significant (29 (42.6%) vs. 72 (31.3%), *p* = 0.095).

The cisternography found that the most frequent level of CSF leakage was the thoracic level. The percentages of patients with CSF leakage at the cervical, thoracic, and lumbar levels were 44.3% (*n* =132), 51.3% (*n* = 153), and 21.5% (*n* = 64), respectively. CSF leakage was undetermined in 45 patients (15.1%). Multiple leakage was detected in 160 patients (53.7%). The median CSF opening pressure was 4.8 (0.0–8.0) mmHg. Early bladder activity was detected in 59 patients (19.8%). None of these factors differed significantly in the groups of poor and good responders.

### 3.3. Number, Volume, and Site of Epidural Blood Patch

Among the study group, the maximum number of EBPs was eleven. Poor responders received a median of three (3–4) EBPs, whereas good responders received a median of one (1–2) EBP (Table 3). The mean volume of the first EBP was 15.0 (12.0–15.0) mL, with no significant difference between poor responders and good responders (15.0 (12.0–17.8) mL vs. 15.0 (12.0–15.0) mL, *p* = 0.717). In addition, sites of the first EBP delivery did not differ significantly in the two groups.

### 3.4. Laboratory Data

Table 4 shows the laboratory data in the two groups. The prothrombin time; neutrophil and lymphocyte counts; platelet distribution time width; neutrophil-to-lymphocyte ratio (NLR); and platelet-to-lymphocyte ratio (PLR) differed significantly in poor and good responders. The prothrombin time (international normalized ratio) was significantly higher in poor than in good responders (1.0 ± 0.1 vs. 0.99 ± 0.1, *p* < 0.001), but the difference was not clinically significant. The platelet distribution width (%) was significantly lower in poor than in good responders (11.1 (10.2–12.0)% vs. 11.4 (10.7–12.7)%, *p* = 0.046). The neutrophil percentage was significantly lower (57.8 ± 13.2% vs. 63.0 ± 11.5%, *p* = 0.002) and the lymphocyte percentage significantly higher (33.4 ± 10.7% vs. 28.5 ± 9.9%, *p* = 0.001) in poor than in good responders. Consequently, the NLR was significantly lower in poor than in good responders (1.8 (1.2–2.5) vs. 2.2 (1.6–3.5), *p* = 0.002). In addition, the PLR was also significantly lower in poor than in good responders (117.0 (83.4–145.4) vs. 131.3 (104.0–160.9), *p* = 0.010). The platelet to neutrophil ratio was marginally higher in poor than in good responders (68.1 (44.4–87.6) vs. 58.2 (43.1–77.6), *p* = 0.072), but the difference was not statistically significant.

### 3.5. Hospitalization Period

The average hospitalization period after EBP in all subjects was 7 ± 6 days. The hospitalization period was significantly longer in poor than in good responders (12 ± 7 vs. 5 ± 4 days, *p* < 0.001).

### 3.6. Univariate and Multivariate Logistic Regression Analysis

Univariate logistic regression analysis showed that the NLR and PLR were significantly associated with a poor response to EBP in patients with SIH (Table 5). The consideration of biological plausibility, clinical importance, and statistical difference (*p* < 0.10) resulted in the inclusion of the NLR, the PLR, EBP sites, and the brain engorgement of venous structures in a multivariate logistic regression analysis. After confirming that there was no multicollinearity among these variables, multivariate logistic regression analysis showed that NLR (odds ratio = 0.720, *p* = 0.008) was independently and significantly associated with a poor response to EBP in patients with SIH (Table 5).

## 4. Discussion

In the present study, we comprehensively analyzed related factors associated with EBP treatment outcome in patients with SIH. Comparisons between poor and good responders showed no differences in demographic characteristics, EBP sites and injected blood volumes. Interestingly, laboratory data showed that a low NLR was significantly and independently associated with a poor response to EBP treatment in patients with SIH. Overall, EBP showed good outcomes in these patients without complications.

In intracranial hypotension, CSF leaks induced by various causes at a rate that exceeds CSF production leads to a decrease in CSF volume. Any change in intracranial pressure caused by intracranial hypotension may influence the cerebral blood flow. Most studies revealed the intracranial vessel dilatation, a cerebral blood flow increase, a flow velocity decrease, and cerebral venous thrombosis whereas some studies showed intracranial hypotension associated with vasoconstrictions and flow velocity increases [26].

Intracranial hypotension often does not require aggressive treatment because patients recover spontaneously or recover by conservative managements [27,28]. If conservative management fails to bring symptom relief, EBP can be considered as a first line interventional treatment, whether or not there is explicit CSF leakage [8,29]. Although this interventional management has shown favorable results [28], the response of each EBP is unpredictable and a single attempt may be insufficient [9]. Repeated EBP may therefore be required. This study evaluated factors associated with the response to EBP, which may help estimate the duration of hospitalization, avoid unnecessary EBP, or enhance favorable outcomes.

The main finding of the present study was that a low NLR was independently and significantly associated with a poor response to EBP in patients with SIH. To our knowledge, there have been few reports about the clinical outcome of EBP in SIH. More importantly, this is the first study of the relationship between an inflammatory maker such as NLR and outcome of EBP. The NLR is a marker of systemic inflammation [30,31]. A high NLR in patients with various types of solid cancers is associated with poor overall survival [32]. Moreover, a high NLR may be a prognostic predictor of outcomes in patients with sepsis [33]. The NLR may also be an indicator of outcomes in various clinical conditions, such as preeclampsia, metabolic syndrome, and rheumatoid arthritis [34,35]. Inflammation plays an important role in various clinical circumstances, therefore, the NLR is a useful marker [30,31,32]. In addition, inflammation activate coagulation and coagulation also considerably affects inflammatory activity [36]. Inflammation especially can cause thrombosis by various mechanisms such as molecular pathways, pro-inflammatory cytokines, or a von Willebrand factor-mediated platelet aggregation [36,37]. The effect of EBP is determined as an effect related simply to volume replacement by compressing the dura mater, and a subsequent latent effect related to sealing of the leakage [38]. In terms of the sealing of the leakage site, platelets and coagulation may play an important role related with EBP outcomes, as EBP with platelet rich plasma was successful in treating patients unsuccessfully treated with conventional EBP [39,40]. Our results therefore suggest that a low NLR may hinder the aggregation of platelets, consequently associated with a poor response to EBP.

In the present study, the difference in the NLR between the groups of poor and good responders was relatively smaller than in other studies [32,33,41]. The NLR in patients with SIH can differ from that in patients with serious underlying diseases such as cancer, sepsis, and pulmonary embolism [32,33,41]. However, those patients were critically ill and had high morbidity and mortality, therefore, the NLR of those patients would have been higher than our study. In Hashimoto thyroiditis, the NLR was significantly higher in patients diagnosed with Hashimoto thyroiditis compared with the control group (2.43 ± 0.94 vs. 2.11 ± 0.81; *p* < 0.05) [42]. In patients with obesity, the NLR was 1.68 (1.45–3.14) and it was significantly correlated with pro-inflammatory mediators, adiposity biomarkers, and progressive subclinical atherogenesis [42]. Moreover, in patients with obstructive sleep apnea treated using mandibular advancement devices, the pre-treatment NLR for the study group was 2.71, which is elevated relative to 1.81 reported in the normal population [43]. NLR has not been determined definitively in patients with SIH, although the median NLR in healthy individuals is 1.65 [44]. Therefore, the NLR could be lower in patients with SIH than in those with critical illnesses, resulting in a relatively small difference in the NLR between poor and good responders.

In this study, patients were divided into two groups based on their response to EBP. Poor responders experienced symptom relief after three or more EBPs, whereas good responders consisted of patients who experienced symptom relief after one or two EBPs. A previous study found that only 57% of patients of SIH experienced complete symptom resolution after single EBP and 20% of patients experienced complete symptom resolution after two EBPs [9]. Another study showed that the success rate of each EBP was approximately 30% [24]. In other words, EBP was frequently less successful in patients with SIH than in those with PDPHs and repetitive EBP could be needed in the patients with SIH [8,9]. In this study, sometimes three to eleven EBPs have been given before achieving a lasting relief. Furthermore, radiologic examinations showed that there were one hundred and sixty (53.7%) patients who had at least two different suspected leakage sites and forty-five (15.1%) subjects failed to determine the accurate leakage site. The patients with multiple leakage sites may need more EBP compared to those with a single leakage site. These findings were in line with previous studies [11,25] and suggested that these patients may require more than two separate EBP attempts. Accordingly, poor responders were defined as patients who required three or more EBPs and good responders were defined as patients who required one or two EBPs in our study.

The prevalence of hypertension was lower in poor responders than in good responders. The CSF pressure was increased by angiotensin-induced systemic hypertension in an animal study [45]. Because EBP increased CSF pressure by compressing the dura mater [46], the pressure effect of EBP seemed to be higher in patients with hypertension compared with patients without hypertension. However, there was no significant association with hypertension and the response to EBP in the univariate analysis. Therefore, the relationship between hypertension and the EBP outcome needs to be interpreted cautiously.

We investigated the average hospitalization periods of poor and good responders to EBP. As expected, the hospitalization period was significantly longer in the poor than in the good response group. A poor response to initial EBP required additional EBPs, suggesting that the longer hospitalization period in poor responders represented the natural course of the disease. Patients with SIH and a low NLR may require a longer hospitalization period. This finding may help in planning treatment in patients with SIH and in estimating the required supply of EBP.

This study had several limitations. First, because this study had a retrospective design, we could not evaluate all covariates that may have affected the analysis. Therefore, our study may be, at least in part, influenced by inevitable selection bias. However, we included almost all covariate factors related to the response to EBP in patients with SIH. Second, the platelet count, prothrombin time, and activated partial thromboplastin time were within normal values, although our hypothesis that a low NLR was associated with hindered platelet aggregation and the need for additional EBPs in poor responders has not yet been tested. Platelets have been recognized as potent immune modulators and as playing a role in host defenses against infection [47,48]. Clinical laboratory data related to platelet aggregation, such as the bleeding time, platelet function tests, thromboelastography, or rotational thromboelastometry may be useful in testing our hypothesis. Future studies should investigate the relationship between subclinical inflammation and platelet aggregation function. Third, the definition of a poor and good responder might be arbitrary. However, the aim of this study was to evaluate the factor of a poor response to EBP in patients with SIH. In other studies, 23–30% of patients with SIH have required EBP at least three times [8,9]. Therefore, we defined the poor responder as the patients who required EBP at least three times. Lastly, we did not analyze other neuroimaging factors associated with EBP outcome such as epidural CSF collection, and the degree of iter descent below the incisural line [21].

## 5. Conclusions

A low NLR may be associated with three or more administrations of EBP for the sufficient improvement of symptoms in patients with SIH. The laboratory analysis of blood samples may predict responses and the outcomes of EBP in patients with SIH.

## Figures and Tables

**Table 1:** Demographic and clinical characteristics of patients with spontaneous intracranial hypotension who underwent epidural blood patch.

Variables	Total Patients (*n* = 298)	Poor Responders (*n* = 68)	Good Responders (*n* = 230)	*p*-Value
Age, years	38 (33–46)	38 (34–46)	38 (33–46)	0.952
Sex				
Male/Female, *n* (%)	108 (36.2%)/190 (63.8%)	27 (39.7%)/41 (60.3%)	81 (35.2%)/149 (64.8%)	0.499
Height, cm	164.6 ± 22.3	165.4 ± 8.9	164.4 ± 8.2	0.365
Weight, kg	58.0 (52.3–68.0)	58.5 (52.1–69.0)	58.0 (52.4–67.9)	0.816
Body mass index, kg/m^2^	22.3 ± 2.9	21.9 ± 2.8	22.4 ± 2.9	0.270
Underlying disease				
Diabetes mellitus, *n* (%) Hypertension, *n* (%) Coronary arterial disease, *n* (%) Cerebrovascular accident, *n* (%) Herniated intervertebral disc, *n* (%)	12 (4.0%) 15 (5.0%) 6 (2.0%) 1 (0.3%) 10 (3.4%)	1 (1.5%) 0 (0.0%) 3 (4.4%) 0 (0.0%) 2 (2.9%)	11 (4.8%) 15 (6.5%) 3 (10.3%) 1 (0.4%) 8 (3.5%)	0.310 0.027 0.130 >0.999 >0.999
History of headache				
Migraine, *n* (%) Tension headach, *n* (%) Cluster headache, *n* (%)	11 (3.7%) 3 (1.0%) 0 (0.0%)	5 (7.4%) 0 (0.0%) 0 (0.0%)	6 (2.6%) 3 (1.3%) 0 (0.0%)	0.613
Associated symptoms				
Nausea, *n* (%) Vomiting, *n* (%) Photophobia, *n* (%) Hearing impairment, *n* (%) Tinnitus, *n* (%) Vertigo, *n* (%) Diplopia, *n* (%)	166 (55.7%) 100 (33.6%) 2 (0.7%) 4 (1.3%) 65 (21.8%) 1 (0.3%) 1 (0.3%)	42 (61.8%) 29 (42.6%) 1 (1.5%) 2 (2.9%) 8 (11.8%) 0 (0.0%) 0 (0.0%)	124 (53.9%) 71 (30.9%) 1 (0.4%) 2 (0.9%) 24 (10.4%) 1 (0.4%) 1 (0.4%)	0.194 0.056 0.400 0.220 0.723 >0.999 >0.999
Duration of headache, days	10.0 (9.0–30.0)	15.0 (9.0–30.0)	10.0 (9.0–30.0)	0.579
Headache, numeric rating scale	7.0 (5.0–9.0)	7.0 (4.0–9.0)	7.0 (5.0–8.0)	0.790

Data are expressed as the mean ± standard deviation, median (interquartile range), or number (%). Poor responders, patients who underwent epidural blood patch three or more times; good responders, patients who underwent epidural blood patch one or two times.

**Table 2:** Brain MRI, cisternography, cerebrospinal opening pressure, and early bladder activity in patients with spontaneous intracranial hypotension patients after epidural blood patch.

Variables	Total Patients (*n* = 298)	Poor Responders (*n* = 68)	Good Responders (*n* = 230)	*p*-Value
Brain MRI signs				
Pachymeningeal enhancement, *n* (%) Engorgement of venous structures, *n* (%) Brain sagging, *n* (%) Pituitary hyperemia, *n* (%) Midline shift, *n* (%) Midbrain–pons angle, degrees Vein of Galen–Straight sinus angle, degrees	161 (54.0%) 101 (33.9%) 40 (13.4%) 32 (10.7%) 5 (1.7%) 55.3 ± 10.0 63.4 (46.6–74.1)	35 (51.5%) 29 (42.6%) 7 (10.3%) 7 (10.3%) 0 (0.0%) 55.0 ± 8.8 64.1 (43.0–74.6)	126 (54.8%) 72 (31.3%) 33 (14.3%) 25 (10.9%) 5 (2.2%) 55.3 ± 10.4 63.1 (47.5–73.5)	0.542 0.095 0.367 0.864 0.592 0.800 0.934
Cisternography				
Level of cerebrospinal fluid leakage				
Cervical, *n* (%)	132 (44.3%)	29 (42.6%)	103 (44.8%)	0.755
Thoracic, *n* (%)	153 (51.3%)	37 (54.4%)	116 (50.4%)	0.564
Lumbar, *n* (%)	64 (21.5%)	19 (27.9%)	45 (19.6%)	0.140
Undetermined, *n* (%)	45 (15.1%)	6 (8.8%)	39 (17.0%)	0.100
Multiple leakage, *n* (%)	160 (53.7%)	43 (63.2%)	117 (50.9%)	0.159
Cerebrospinal opening pressure, mmHg	4.8 (0.0–8.0)	4.5 (0.0–7.5)	5.0 (0.0–8.2)	0.580
Early bladder activity, *n* (%)	59 (19.8%)	17 (25.0%)	42 (18.3%)	0.369

Data are expressed as the mean ± standard deviation, median (interquartile range), or number (%). Poor responders, patients who were delivered epidural blood patches more than three times; good responders, patients who were delivered epidural blood patches one or two times.

**Table 3:** Number, volume, and sites of epidural blood patch in patients with spontaneous intracranial hypotension.

Variables	Total Patients (*n* = 298)	Poor Responders (*n* = 68)	Good Responders (*n* = 230)	*p*-Value
Number of epidural blood patches, times	2 (1–2)	3 (3–4)	1 (1–2)	<0.001
First epidural blood patch volume, mL	15.0 (12.0–15.0)	15.0 (12.0–17.8)	15.0 (12.0–15.0)	0.717
First epidural blood patch sites				
Cervical, *n* (%) Thoracic, *n* (%) Lumbar, *n* (%)	130 (43.6%) 138 (46.3%) 30 (10.1%)	21 (30.9%) 40 (58.8%) 7 (10.3%)	109 (47.4%) 98 (42.6%) 23 (10.0%)	0.062

Data are expressed as the mean ± standard deviation, median (interquartile range), or number (%). Poor responders, patients delivered epidural blood patches at least three times; good responders, patients delivered epidural blood patches one or two times.

**Table 4:** Laboratory data in patients with spontaneous intracranial hypotension after epidural blood patch.

Variables	Total Patients (*n* = 298)	Poor Responders (*n* = 68)	Good Responders (*n* = 230)	*p*-Value
Hemoglobin, g/dL	13.6 ± 1.6	13.8 ± 1.3	13.6 ± 1.7	0.430
Hematocrit, %	40.3 ± 4.0	40.6 ± 3.6	40.2 ± 4.2	0.386
Platelet, 10^3^/μL	241.2 ± 55.6	238.8 ± 48.8	241.9 ± 57.5	0.694
Prothrombin time, INR	1.0 ± 0.1	1.0 ± 0.1	0.99 ± 0.1	<0.001
Platelet distribution width, %	11.3 (10.6–12.5)	11.1 (10.2–12.0)	11.4 (10.7–12.7)	0.046
Activated PTT, sec	27.5 (26.0–29.1)	27.7 (26.2–29.0)	27.4 (26.0–29.1)	0.429
White blood cell, cells/mm^3^ Neutrophil, % Lymphocyte, % Monocyte, % Eosinophil, % Basophil, %	6600 (5400–8100) 61.8 ± 12.1 29.6 ± 10.3 6.2 (4.9–7.4) 1.4 (0.6–2.4) 0.3 (0.2–0.5)	6500 (5100–8000) 57.8 ± 13.2 33.4 ± 10.7 6.6 (5.1–7.6) 1.4 (0.6–2.5) 0.3 (0.2–0.5)	6600 (5500–8200) 63.0 ± 11.5 28.5 ± 9.9 6.1 (7.4–4.9) 1.4 (0.6–2.4) 0.37 (0.2–0.5)	0.357 0.002 0.001 0.237 0.923 0.904
Absolute neutrophil count, cells/μL	3925 (3000–5552)	3820 (2735–5262)	4020 (3040–5632)	0.266
Erythrocyte sedimentation rate, mm/hr	9.0 (4.0–18.0)	7.5 (3.3–15.8)	10.0 (4.0–18.0)	0.209
C-reactive protein, mg/L	0.1 (0.1–0.1)	0.1 (0.1–0.1)	0.1 (0.1–0.2)	0.215
Neutrophil-to-lymphocyte ratio	2.1 (1.5–3.2)	1.8 (1.2–2.5)	2.2 (1.6–3.5)	0.002
Platelet-to-lymphocyte ratio	129.2 (100.5–158.5)	117.0 (83.4–145.4)	131.3 (104.0–160.9)	0.010
Platelet-to-neutrophil ratio	60.8 (43.3–81.5)	68.1 (44.4–87.6)	58.2 (43.1–77.6)	0.072

Data are expressed as the mean ± standard deviation, or median (interquartile range). Poor responders, patients who underwent epidural blood patch at least three times; good responders, patients who underwent epidural blood patch one or two times; neutrophil-to-lymphocyte ratio, the ratio of the absolute neutrophil counts and the absolute lymphocyte counts; platelet-to-lymphocyte ratio, the ratio of the absolute platelet counts and absolute lymphocyte counts; platelet-to-neutrophil ratio, the ratio of the absolute platelet counts and absolute neutrophil counts; PTT, partial thromboplastin time; INR, international normalized ratio.

**Table 5:** Logistic regression analysis of factors associated with poor response to epidural blood patch.

Variables	Univariate			Multivariate		
	OR	95% CI	*p*-Value	OR	95% CI	*p*-Value
Hypertension	0.000	0.000–NC	0.998			
Neutrophil-to-lymphocyte ratio	0.705	0.556–0.894	0.004	0.720	0.565–0.917	0.008
Platelet-to-lymphocyte ratio	0.992	0.986–0.998	0.015	0.996	0.989–1.004	0.370
Epidural blood patch site						
Cervical	1			1		
Thoracic	0.633	0.241–1.664	0.354	0.479	0.174–1.323	0.156
Lumbar	1.341	0.533–3.474	0.533	1.052	0.401–2.761	0.918
Brain engorgement of venous structures	1.611	0.919–2.826	0.096	1.679	0.931–3.028	0.085

Poor responders were defined as patients who were delivered epidural blood patches three or more times. Neutrophil-to-lymphocyte ratio, the ratio of the absolute neutrophil counts and the absolute lymphocyte counts; platelet-to-lymphocyte ratio, the ratio of the absolute platelet counts and absolute lymphocyte counts; OR, odds ratio; CI, confidence interval; NC, not calculated.
